# Recent Advancement in the Management of Intrauterine Adhesions Using Stem Cell Therapy: A Review Article

**DOI:** 10.7759/cureus.43553

**Published:** 2023-08-16

**Authors:** Shreya Parashar, Sandhya Pajai, Tanmay Tarang

**Affiliations:** 1 Obstetrics and Gynecology, Jawaharlal Nehru Medical College, Datta Meghe Institute of Medical Sciences, Wardha, IND

**Keywords:** uterine regeneration, newer treatment modality, stem cells therapy, intrauterine adhesions, asherman’s syndrome

## Abstract

Intrauterine adhesions (IUAs) are the formation of scar tissues in the endometrial cavity. The fibrous tissue in the uterus decreases the space inside the uterine cavity. It includes both endometrium and myometrium. It may lead to hypomenorrhea or amenorrhea, pain, difficulty in conceiving, and recurrent abortion. IUA is caused by uterine tissue damage mostly during surgical procedures such as dilatation and curettage. Other causes may include pregnancy-related complications, miscarriage, abnormal bleeding, infections, fibroid removal, and cesarean section (C-section). Patients generally do not have any symptoms and hence are unaware of the condition. The main therapeutic procedure presently used is hysteroscopic transcervical resection of adhesion (TCRA) with hormonal therapy and nondegradable stent as postoperative adjuvant therapy. It has some major limitations such as failure to prevent recurrence and preserve fertility along with difficulty in endometrial tissue repair due to its anatomical site. These limitations have forced the researchers to think about a better treatment modality. In recent times, a better treatment modality has evolved with stem cell therapy. Therefore, this review presents the recent and advanced therapeutic modalities for the treatment of IUAs.

## Introduction and background

Intrauterine adhesions (IUAs) also called Asherman’s syndrome is one of the most severely undiagnosed conditions among women. It is caused as a result of trauma to the endometrial tissue resulting in hypomenorrhea or amenorrhea, recurrent abortion, and infertility [1]. It could be primary or secondary. Primary adhesions take place as a result of pregnancy-related curettage or posthysteroscopic surgery, whereas secondary adhesions commonly occur following postadhesiolysis reoccurrence [2,3]. It could be asymptomatic or symptomatic. When asymptomatic, the term Asherman's syndrome is avoided [1]. Disturbed menstrual cycle, recurrent abortion, or failure to conceive is the most common presenting symptoms of the patients. Factors that damage the endometrium can cause adhesion of myometrium at the opposing wall and decreases the intrauterine cavity space [4]. Some of these factors are trauma after pregnancy, trauma without pregnancy, and genital tuberculosis [4]. Trauma after pregnancy includes curettage after abortion, postpartum curettage, and C-section, whereas trauma without pregnancy includes myomectomy, diagnostic curettage, insertion of intrauterine devices (IUDs), and hysterectomy [4]. It has a high prevalence in Israel, Greece, France, South America, the United States, and Japan due to unknown reasons [4]. Due to such irregular distribution, no geographical factor is responsible for it. Knowledge about the pathophysiology of Asherman's syndrome and IUA is limited. Many theories about it have been put forward from time to time. High impendence of the spiral artery and disturbed vascularity of the myometrium and endometrium due to endometrial damage explain the decreased regeneration and receptivity of the endometrium in these women [5]. Mitochondrial swelling, hypoxic cellular modification, and ribosome loss are some of the cellular changes seen in this condition [6]. Cytokines responsible for the pathogenesis are basic fibroblast growth factor (FGF-B), platelet-derived growth factor (PDGF), and transforming growth factor type 1 (TGF-1) [7]. For proper management and good prognosis, classification was required, which was done by March et al. in 1978, as cited in [8], and is still used for its simplicity, as shown in Table 1.

**Table 1:** Classification of intrauterine adhesion. Source: [8].

Serial number	Classification	Condition
1	Mild	Occupy lesser than one-fourth cavity space with upper fundus and ostial area clear.
2	Moderate	Ostial areas and upper fundus are not completely involved, and up to one-third of the cavity is damaged.
3	Severe	More than one-third cavity involved with ostial area and upper fundus occluded and agglutination of the uterine wall.

Mostly being asymptomatic, the diagnosis accidental. The new endoscopic procedure that has enabled easy access inside the uterine cavity has made diagnosis and management very easy. Hysteroscopy is the gold standard diagnostic modality [9]. It can also be diagnosed with the help of magnetic resonance imaging (MRI), three-dimensional (3D) ultrasonography (USG), contrast ultrasonography, and hysterosalpingography [9]. With the development of the hysteroscopic technique, transcervical resection of adhesion (TCRA) is the main standard treatment, but it has its limitations. High recurrence rate and inability to repair endometrium are its most common limitations [10]. Stem cell therapy is the newer treatment modality that has been discovered in recent times, which has overcome the limitations of transcervical resection of adhesion [10].

Stem cells are completely or partially undifferentiated cells that can differentiate into a particular type of cell. They can be cultured in long-term in vitro. They have cell surface markers and in vivo reconstruction capacity. Embryonic stem cells and adult stem cells are the two types of stem cells [11]. Embryonic stem cells have a high proliferation rate as well as have the potential of changing into malignancy, whereas adult stem is less tumorigenic. Hence, adult stem cells are mostly used for therapeutic uses [11]. The endometrium has a great capacity for regeneration. After delivery and menstruation, the endometrium regenerates itself normally. The epithelial progenitor cells and mesenchymal cells in the endometrium can be used to regenerate and repair the endometrial tissue of the uterus [12]. According to recent studies, a patient’s bone marrow has also been proven a source of stem cell production [13].

IUAs are one of the most undiagnosed and underdiagnosed conditions and have only limited treatment modalities. These treatment modalities have limitations that are responsible for the poor prognosis of this condition. Newer treatment modalities are under research; therefore, this review provides detail about the new treatment modality that may overcome the drawbacks of the present treatment technique.

## Review

**Figure 1:**
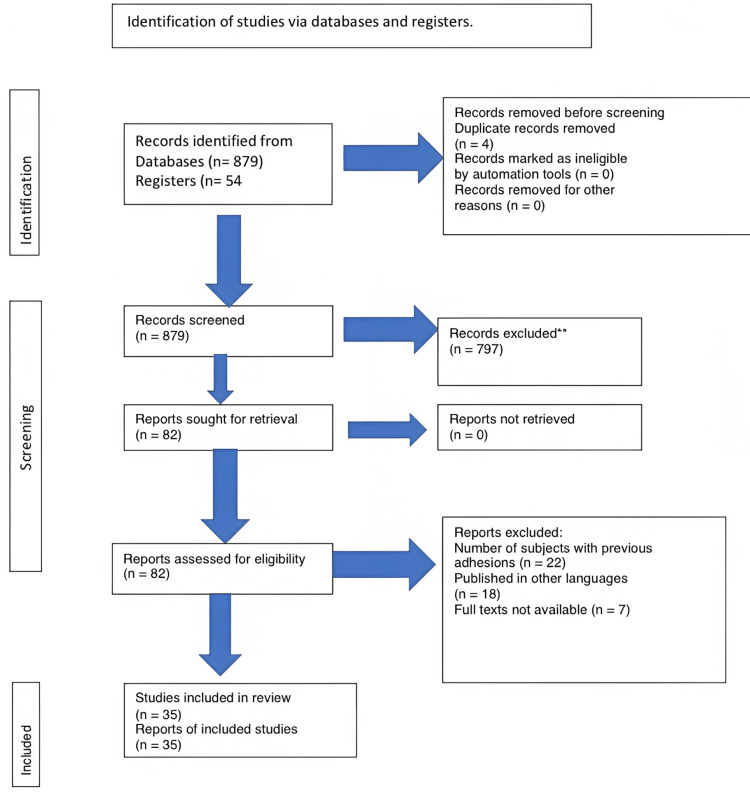
PRISMA flow diagram for the selection of data required for the review. Image credit: Shreya Parashar. PRISMA, Preferred Reporting Items for Systematic Reviews and Meta-Analyses

We undertook a systematic search through PubMed and CENTRAL in November 2022 using keywords such as "Asherman syndrome" and "stem cell therapy" (((Asherman syndrome [Title / Abstract) OR (stem cell therapy (Title / Abstract])) OR (koch*[Title / Abstract])) OR ("Asherman syndrome" [MesH terms|) AND (("stem cell therapy" [Title / Abstract) OR (ScT [Title/ Abstract])) OR ("stem cell Therapy" [MesH ferms]). We additionally searched for key references from bibliographies of the relevant studies. the search was updated in February 2023. One reviewer independently monitored the retrieved studies against the inclusion criteria, in the beginning, based on the title and abstract and then on the full text. Another reviewer also reviewed approximately 20% of these studies to validate the inclusion of studies, as shown in Figure 1.

Stem cell therapy in the regeneration of endometrial tissue has shown great results in both animal and human models [9]. Different delivery methods such as infusion in spiral arterioles, direct implantation of the stem cells in the endometrial cavity, and subendometrial area via transmyometrium have been used so far [14].

**Figure 2:**
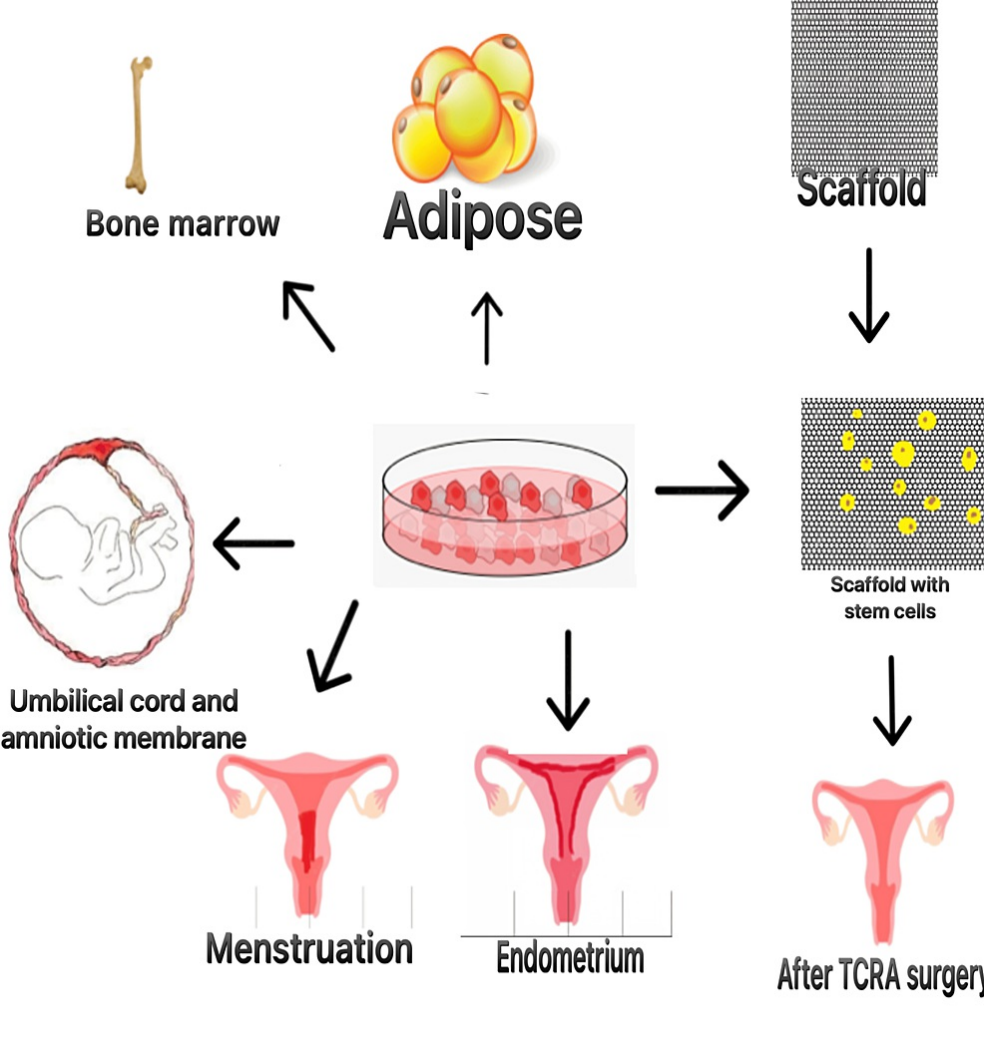
Sources of stem cells and mechanisms of use in TCRA surgery. Image credit: Shreya Parashar. TCRA, transcervical resection of adhesion

Stem cell transplantation has many therapeutic approaches toward tissue repair and hence is an excellent alternative form of treatment. Recently, postoperative stem cell therapy is a great approach to resolving reproduction-related abnormalities in IUA patients. The uterine sources of stem cells are mesenchymal stem cells (MSCs)/stromal cells, epithelial stem cells (ESCs), and endothelial stem cells. All of these cells have the capacity for endometrial repair [15]. Nonuterine sources of stem cells production are induced pluripotent stem cells (iPS), human embryonic stem cells (hES), bone marrow-derived stem cells (BMDSC), and umbilical cord-derived MSCs (UC-MSCs) [16-18]. Various sources of stem cells are shown in Figure 2.

Endogenous stem cells

Mesenchymal Stem Cells

MSCs act as pericytes and perivascular cells and are seen on the outer surface of the small blood vessels that promote endometrial cell regrowth, maturation, and hemostasis [19]. Properties of MSCs are classified into three criteria as stated by the International Society for Cellular Therapy (ISCT), as shown in Table 2 [20]. Menstrual blood-derived MSC-like cells and human endometrial CD-146+ and CD-140+ cells, and other types of MSCs have similar gene profiles in immunomodulatory potential and low immunogenicity.

**Table 2:** Properties of mesenchymal stem cells. Source: [20]. CD, cluster of differentiation; HLA, human leukocyte antigen

Properties	Characteristics
Growth property	Must be plastic-adherent in standard culture conditions
Surface marker	Must express cluster of differentiation (CD)105, CD90, and CD73 and not able to express CD14, CD34, CD45, CD19, CD79α, CD11b, and HLA-DR surface molecules
Differentiation potential	Capable of definite differentiation into osteoblasts, chondroblasts, and adipocytes in vitro

CD140b also called platelet-derived growth factor receptor-β, CD146, stage-specific embryonic antigen (SSEA-1), and leucine-rich repeat-containing G-protein (LGR5) are some of the specific MSC markers [17]. These markers are used for detecting MSCs in the transplanted tissues.

Epithelial Stem Cells

ESCs are found in the basalis layer of the endometrium and are not shed off during menstruation. These cells are large, single-cell-derived colony forming units (CFUs) and have high proliferating capacity [21]. These cells can be differentiated into big gland-like structure through 3D cell culture [21]. Generation of gland-like structure in vitro is done by nuclear SOX9+ epithelial cell sand SSEA 1+ (SSEA 1+) [22]. SSEA+ has great telomerase activity. SSEA-1+ cells show greater telomerase activity in comparison with SSEA-1- epithelial cells along with longer telomeres and lower proliferation rates, which define the characteristics of progenitor cells. N-cadherin+ ESCs are found on the base of the endometrial gland at the interface formed by the endometrium/myometrium. After a thorough analysis of the special relationship of SSEA-1+ ESC and N-cadherin+ ESC, it can be observed that there is an obvious epithelial differentiation in the endometrial glands epithelia.

Endothelial Stem Cells

The endometrium cells have two types of populations, main populations and side populations. The endometrial side population has a great clonogenic capacity but lacks a niche. The endometrial main population lacks clonogenic capacity but has a great niche. When both EMP and ESP are co-cultured, they show great success in forming uterine environment [23].

Exogenous stem cells

Bone-Marrow-Derived Stem Cells

Stem cells from the bone marrow can differentiate into multiple nonhematopoietic cell lineages. Studies have suggested endometrial cells and stromal cells can be formed from donor-derived bone marrow cells [18]. The tissue regeneration takes place via engraftment, production of cytokines, recruitment of other cells, and vigorous differentiation of the bone marrow stem cells that migrate to the injured site. Hypoxia, chemotherapy, and irradiation stimulate the recruitment of the stem cells.

*Umbilical Cord MSC* *s*

UCMSCs have a high cartilage differentiation rate and high proliferative capabilities [24]. They are also easily available and have low immunogenicity. It is located in the Wharton jelly of the umbilical cord and around the blood vessels [24]. UCMSCs decrease the apoptosis of stromal tissue and increase the proliferation of healthy tissues.

Genes for Endometrial Regeneration

Wingless-related integration site (Wnt), receptor tyrosine kinase (c-kit) (CD117), octamer-binding transcription factor-4 (Oct-4), CD34, and Musashi-1 are some of the many genes/proteins that are responsible for the complete regeneration of the endometrium. A signal cascade regenerated by the Wnt gene secretes a protein that binds to the surface marker of the frizzled family and decides the future of the cells. Wnt7a is responsible for the regeneration of endometrium, and progesterone suppresses it in the latter half of the menstruation and causes the epithelium to secretory phenotype transformation [25]. Müllerian duct formation, differentiation, and regression are also regulated by the Wnt gene [26]. Proliferation and stromal decidualization during embryo implantation and growth as well as internal genitalia development in mice is controlled by Wnt4.

Mesenchymal to Epithelial Transition

Stem cells have a unique feature of cellular transdifferentiation where one somatic cell differentiates from another without going into any intermediate pluripotent state. This feature plays a great role in the repair of the endometrium. Three periods in women’s life where uterine regeneration is significant are embryogenesis, menstruation, and postpartum [26]. Within the stroma lies a unique population of pan-cytokeratin-expressing cells. These cells are seen to relocate to the luminal interface and re-form luminal epithelial during endometrial regeneration.

Immunomodulatory and Antimicrobial Effects of Stem Cells

A dysregulated inflammatory response to an infection causes a life-threatening condition called sepsis. Delays in therapy lead to increased mortality as well as irreparable damage to numerous organs owing to reduced oxygen levels in the tissues and lack of blood flow. Numerous researches have shown a report on the immunomodulatory, anti-inflammatory, anti-apoptotic, and anti-microbial capabilities of MSCs. Therefore, the application of MSCs has been considered a very promising and productive strategy therapeutically. MSCs prevent the growth of B- and T-cells and their development into pro-inflammatory Th1 and Th17 cells as well as *tolerogenic* Treg cells, which are suppressor T-cells that downregulate effector T-cells, in the adaptive system [27]. Instead, MSCs release a wide range of growth-promoting cytokines and signaling molecules, including insulin-like growth factor (IGF-1), transforming growth factor-beta (TGF-b), FGF-B, hepatocyte growth factor (HGF), interleukin (IL-6), stromal-cell-derived (vascular endothelial growth factor [VEGF]), placental growth factor (PIGF), and monocyte chemoattractant protein (MCP-1) [27]. When immune cells are co-cultured with MSCs, they induce type 1 T-helper (Th1) and natural killer (NK) cells to secrete less interferon (IFN-1) and more interferon (IL-4), mature type I dendritic cells (DCs) to secrete less tumor necrosis factor (TNF) and type II DCs to secrete more IL-10, and they cause an increase in the number of Treg cells [28]. Stem cells repair endometrial damage mainly through their paracrine activity, differentiation ability, and immunomodulatory effects.

In Situ Uterine Stents

The application of in situ intrauterine stents can avoid adhesion formation by separating the injured endometrium as well as works as a vehicle for delivering stem cells in various therapies [29]. Artificial scaffolds are smart polymeric nanosystems that can mimic the structural, morphological, and functional properties of the surrounding tissues. Some of the scaffolds that are used are polymer-based hydrogels, polysaccharide-based hydrogels, protein-based hydrogels, and cellular component complex materials.

Polymer-based hydrogels: Many polymers have been studied and used as a treatment for IUAs; some of them are Pluronic F-127, ascorbyl palmitate (AP), polyethylene glycol (PEG), poly-lactic-co-glycolic acid (PLGA), and heparin poloxamer (HP). These polymers can encapsulate many components like growth factors, drugs, stem cells, etc. [30]. One such polymer is Pluronic F-127, which can carry bone marrow stem cells and vitamin C, which are used for the restoration of damaged endometrium due to IUAs [31].

Polysaccharide-based hydrogels: Hyaluronic acid gel (HA-GEL) is one of the most promising polysaccharide-based hydrogels. It is loaded with umbilical cord-derived MSCs that reconstruct and regenerate the endometrium [32].

Protein-based hydrogels: Collagen is one of the most used protein-based biomaterials due to its abundant bioavailabilities. UCMSCs are encapsulated with collagen scaffold and are used in case of recurrence of IUAs postadhesiolysis surgery [33].

## Conclusions

Asherman syndrome is one of the most underrated gynecological problems with very common etiologies. It is mostly caused by postoperative complications. It remains undiagnosed as most of the time it is asymptomatic. There is no appropriate treatment for it without any limitation. TCRA is the most widely used treatment modality but it has its limitation such as recurrence and nonrestoration of the endometrium. With advancing technologies, many newer therapies are under research for it. One such treatment modality is stem cell therapy. It has shown wonderful results in animal and human models. Exogenous and endogenous stem cells along with gene modulation and intrauterine stents are some of the units of stem cell therapy. With these advancing technologies and researches stem cells may fulfill the need of the hour in this condition.
